# Proper hepatic artery transection followed by blunt abdominal trauma: A case report and review of literature

**DOI:** 10.1002/ccr3.9097

**Published:** 2024-06-23

**Authors:** Reyhaneh Naseri, Fatemeh Alishavandi, Mohammad Sadegh Rajabian, Hossein Abdolrahimzadeh Fard

**Affiliations:** ^1^ Thoracic and Vascular Surgery Research Center Shiraz University of Medical Science Shiraz Iran; ^2^ School of Medicine Shiraz University of Medical Sciences Shiraz Iran; ^3^ Student Research Committee, School of Medicine Shiraz University of Medical Sciences Shiraz Iran; ^4^ Department of Surgery Shiraz University of Medical Sciences Shiraz Iran; ^5^ Trauma Research Center, Rajaee (Emtiaz) Trauma Hospital Shiraz University of Medical Sciences Shiraz Iran

**Keywords:** abdominal injuries, avulsion, blunt trauma, hepatic artery, proper hepatic artery

## Abstract

Abdominal vascular injuries, especially in the celiac trunk, are uncommon in blunt trauma, yet their life‐threatening nature necessitates rapid surgical interventions to control possible massive bleedings. Damage control surgery principles may aid management. It is crucial to thoroughly assess all trauma patients, even in instances of initial normal physical examinations.

## 1 INTRODUCTION

Abdominal visceral vessel injuries are scarce but pose significant life‐threatening risks, predominantly associated with penetrating trauma, with only 5%–10% resulting from blunt mechanisms.^1^ The most concerning aspect of abdominal visceral trauma is the potential for up to a 90% mortality rate, often within the initial 24 h due to massive blood loss and consecutive hemorrhagic shock.^2^ Involvement of the celiac artery has been described only in 1%–2% of blunt trauma injuries, and to our knowledge, damage to other celiac branches following blunt trauma has only been documented in case reports.^3^, ^4^ Additionally, Porta hepatis injuries, whether penetrating or blunt, are extraordinarily rare, comprising approximately 0.07% of abdominal vascular injuries. Notably, within this already uncommon group, extrahepatic artery injuries, involving the hepatic artery, are found in just 23% of cases.^5^ Regardless of the rarity of celiac branch injuries resulting from blunt trauma, documenting, and analyzing such cases becomes fundamental, considering their high mortality rates. We aimed to contribute to the current understanding of this condition by describing an unusual case of blunt abdominal trauma that subsequently led to a proper hepatic artery (PHA) abruption.

## 2 CASE PRESENTATION

### 2.1 Case history/examination

A 25‐year‐old male who sustained multiple blunt injuries in a car versus car accident was brought to our emergency department. His past medical history was unremarkable, and he did not take any regular medication. On admission, his physical examination revealed an awake, agitated, and responsive man with a patent airway tract and normal breathing sounds. The vital signs were as follows: a temperature of 37.7°C, blood pressure of 149/109 mmHg, heart rate of 97 beats/min, respiratory rate of 20 breaths/min without respiratory distress, and a maximal Glasgow Coma Score (GCS) of 15. There was no tenderness, rebound tenderness, or guarding on abdominal examination. No lacerations, ecchymoses, or deformities were identified on the chest or abdominal wall and extremities. The remainder of his physical examination was unremarkable. With a history of agitation, the reliability of history‐taking was uncertain. Primary bedside extended focused assessment with sonography for trauma (E‐FAST) was negative and revealed no sign of potentially life‐threatening intra‐abdominal or peri‐hepatic fluid collections or pathology. Initial laboratory investigations found a hemoglobin (Hb) level of 14.9 g/dL and a white blood cell count of 21,300/μL. The venous blood gases measured a pH of 7.27, PCO_2_ of 41.9, and HCO_3_ of 19 with a base deficit of 7.5. The coagulation panel, electrolytes, and creatinine were within normal limits.

**FIGURE 1:**
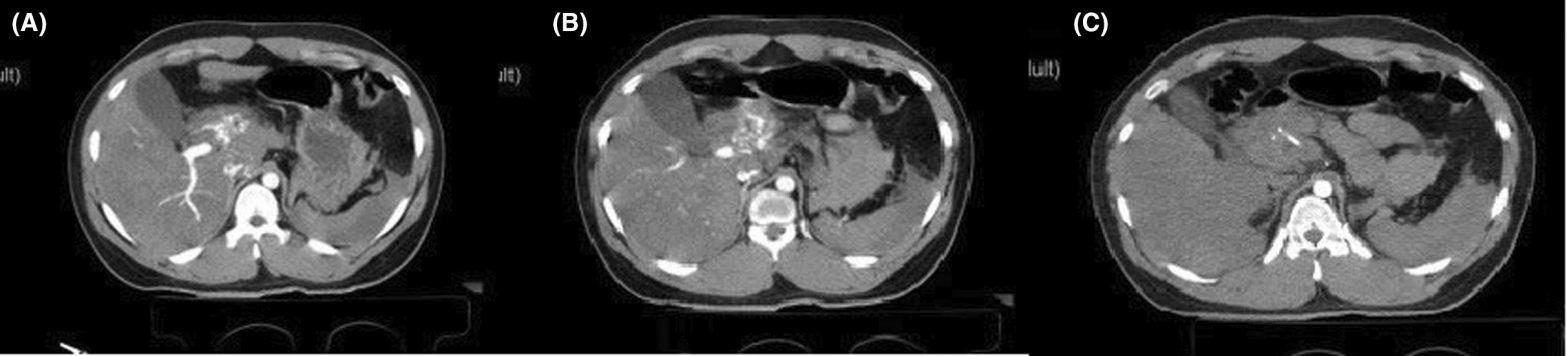
Axial contrast‐enhanced computed tomography images acquired showing contrast material extravasation in the porta hepatis area. (A, B) The dual‐phase (portal‐venous and arterial phase) image demonstrates extravasation of contrast material in the porta hepatis area and moderate free fluid in the abdominal cavity. (C) The arterial phase reveals evidence of hematoma formation in the porta hepatis area around the proper hepatic artery. Free fluid accumulated in spaces adjacent to the spleen is observed on the mentioned plains of the CT scan.

Following the pelvic x‐ray, circumferential pelvic wrapping with a sheet was performed. A chest x‐ray revealed no life‐threatening abnormalities. Intravenous contrast‐enhanced computed tomography (CT) of the abdomen was performed within 2 h after his hospital admission, which showed a hematoma in the porta hepatis with extravasation of contrast material. There were signs of moderate free fluid in the abdominopelvic cavity and small hematoma formation in the pelvic cavity. A left acetabulum fracture was also seen. No solid organ injury was found on imaging (Figure 1).

### 2.2 Differential diagnosis, investigations, and treatment

**FIGURE 2:**
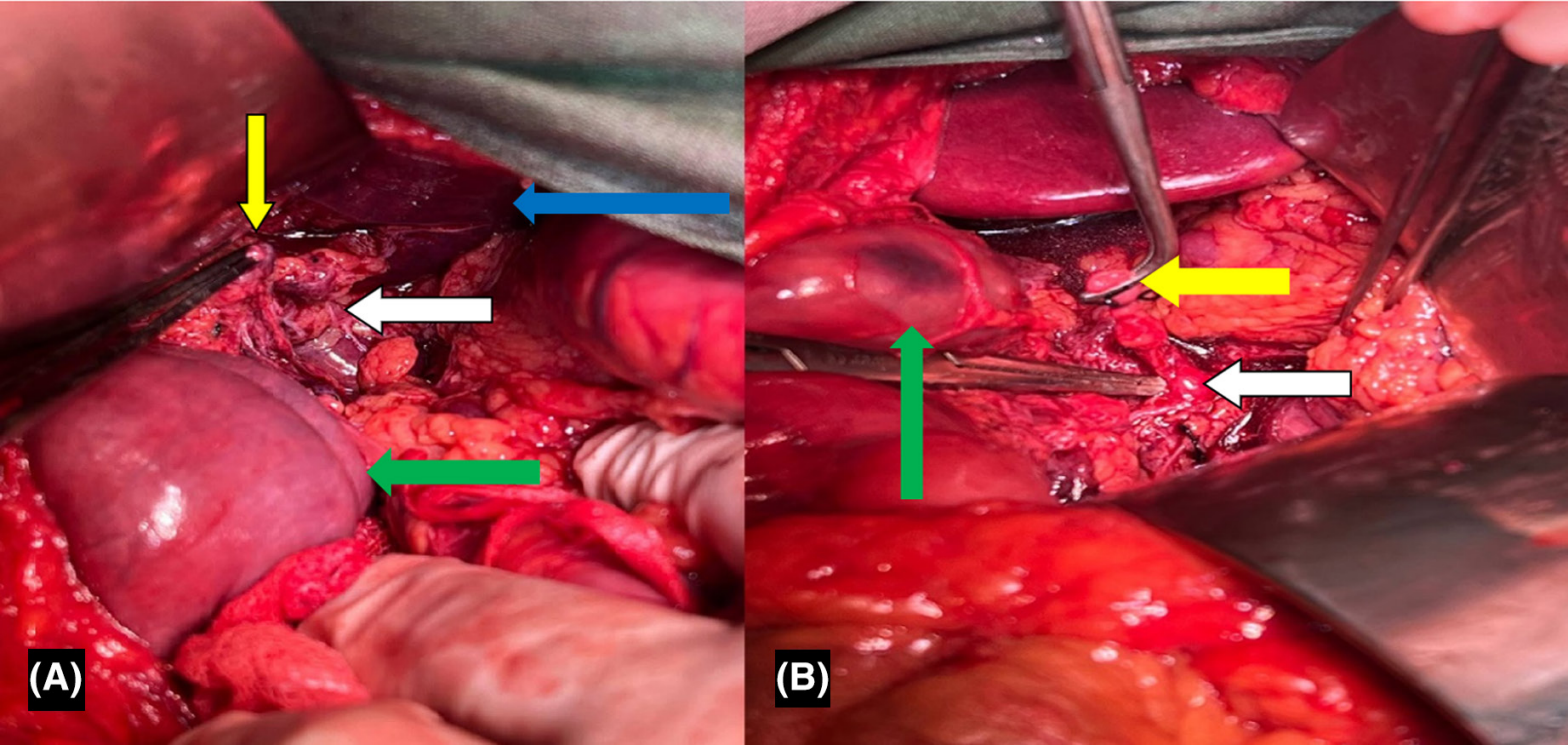
Intraoperative photo of a 25‐year‐old male who sustained multiple blunt injuries in a car versus car accident. (A) The yellow arrow shows the disrupted proper hepatic artery, the white arrow indicates the common bile duct, and the green displays the hepatic flexure of the transverse colon. The blue arrow represents the liver and has been addressed for a better three‐dimensional visualization. (B) The yellow arrow specifies the disrupted common hepatic artery, the white arrow identifies the celiac trunk, and the green arrow labels the duodenum with contusion.

Subsequently, the patient's GCS deteriorated, and his vital signs became unstable, with a palpable central artery (femoral and carotid) pulse rate of 110 beats/min and systolic blood pressure (SBP) of approximately 70 mm Hg. Consequently, one bag of uncrossed packed red blood cells (PC) was requested, and the patient was transferred to the operation room for explorative laparotomy. Simultaneously, a central venous catheter via the right internal jugular vein was inserted for a probable PC transfusion requirement. The patient underwent an exploratory laparotomy via midline incision which revealed approximately 500 cc of free fluid (bloody content) in the abdominal cavity. Initial control of bleeding was achieved with temporary four‐quadrant abdominal packing. Abdominal packs were removed, and further exploration of the abdomen revealed a massive hematoma in the porta hepatis area, followed by fresh bleeding from the lesser sac into the porta hepatis area. Spontaneous bleeding from the lesser sac raised a high key of suspicion for possible celiac trunk injury. Due to SBP <70 mm Hg, digital pressure on the supra‐celiac abdominal aorta was performed by the surgeon. Hence, a left medial visceral rotation maneuver was done. By division of the left crus of the diaphragm in the two o'clock position, full exposure of the proximal abdominal aorta was obtained to allow the visualization of possible injuries to the proximal of the celiac artery. Cross‐clamping of the supra‐celiac abdominal aorta was done using an atraumatic vascular clamp to minimize the bleeding. After extensive dissection and exploration, total avulsion of the PHA just distal to the gastroduodenal artery (GDA) bifurcation was observed. GDA was distally disrupted, which was properly ligated. The common hepatic artery was intact. Intravascular shunt insertion in PHA failed. His secondary laboratory study results were ready in the meantime and showed Hb of 5.3 g/dL, and venous blood gas revealed a pH of 7.14, PCO_2_ of 65.1, and HCO_3_ of 22.1 with a base deficit of 15. Frequent spontaneous massive internal bleeding (approximately 4 L), instability despite adequate fluid substitution, massive transfusion protocol, and persistent low BP (SBP <70 mmHg) despite initiation of high dose norepinephrine infusion throughout the surgery pushed the attending surgeons to decide to ligate PHA as a part of damage control surgery (DCS) in trauma (Figure 2).

Further discoveries in the exploration resulted in an iatrogenic injury to the distal of the pancreas and splenic capsule rupture, leading to distal pancreatectomy and splenectomy. The disrupted superior mesenteric vein (SMV) was ligated. Superior mesenteric artery maintained flow. Right medial visceral rotation was performed to visualize inferior vena cava injuries and ligate the damaged branches. Retroperitoneal hematoma in zone 2 was observed. A grade 1 laceration was detected on the surface of the liver's right lobe near the falciform. Temporary gauze packs were applied, and the rest of the exploration was carried out. Fluid leakage from surrounding tissues of the intact ureter was discovered, which was followed by direct retroperitoneal packing. The abdominal wall was temporarily closed.

### 2.3 Outcome and follow‐up

A transplant surgeon for the reconstruction of the PHA was attended to surgery to prevent abrupt reduction of hepatic arterial blood flow. In the meantime, cardiac arrest occurred, and unfortunately, our patient did not respond to resuscitation measures and could not survive.

## 3 DISCUSSION

### 3.1 Summary of findings and review of current literature

Injuries to abdominal visceral arteries are predominantly caused by penetrating traumas.^2^ While porta hepatis injuries, whether penetrating or blunt, are extraordinarily rare, they can be fatal.^5^ In this study, we presented an exceptional case of PHA abruption caused by blunt trauma to the abdomen in a 25‐year‐old healthy male in the absence of abdominal pain and tenderness and negative FAST results. We believe that our report, among all others, could add to the body of evidence regarding the management of this rare entity.

Transection of the hepatic artery and its branches following blunt trauma is among the least probable vascular injuries reported. In cases of blunt hepatic trauma, pseudoaneurysm formation is the most frequent complication, while both occlusion and transection of the hepatic artery and its branches are significantly less common.^6^ Owing to its rarity, our literature search of English articles in the PubMed database revealed a total of eight cases of hepatic artery transection due to blunt trauma, the features of which are summarized and compared with the present case in Table 1. Since these incidences were only described in case reports, documentation bias is probable, where only severe or interesting cases are reported. Following the predominance of the male population in blunt abdominal trauma, at least 62.5% of our reviewed subjects were male, and the average age was 25.5 (SD = 14.9).^7^ The left hepatic artery was injured in 62.5% of the cases, and our report was the only case of PHA avulsion. Unfortunately, we were unable to retrieve the full article of Graham et al.^8^ Still, among the six remaining patients, five suffered from associated injuries, and our patient was the only case with negative FAST and initial hemodynamic stability. Arterial ligation was the treatment of choice in five cases; however, radiological approaches after hemodynamic stabilization showed promising outcomes in two patients.^9^, ^10^ In a review of 118 porta hepatis injuries, the overall mortality rate was 51%–80%, and 18% of deaths resulted from cardiac arrest in the first 48 h; despite this, the mortality rate in our review was relatively lower, and only one patient (12.5%) passed away.^5^

**TABLE 1:** Clinical and demographical features of patients with hepatic artery transection after a blunt trauma based on a review of published literature.

No.	Author (year)	Gender/age (years old)	Mechanism of injury	Involved artery	Associated injury	Hemodynamic situation on admission	FAST report	Abdominopelvic CT report	Treatment approach	Complications	Outcome
1	Graham et al. (1997)^8^	N/A	N/A	Left hepatic artery	N/A	N/A	N/A	N/A	Open surgery/ligation	Multiple organ failure	N/A
2	Graham et al. (1997)^8^	N/A	N/A	Left hepatic artery	N/A	N/A	N/A	N/A	Open surgery/ligation	N/A	N/A
3	Ripley et al. (2006)^11^	Male/26	Fell from a fifth‐story balcony	Left hepatic artery	Left femur fracture	Unstable/stabled with resuscitation	+	Hemoperitoneum from celiac axis into porta hepatis/extravasation of contrast in celiac axis or portal triad	Open surgery/ligation	N/A	N/A
4	Miglietta et al. (2006)^12^	Male/11	Car versus bicycle accident	Left hepatic artery	Rib fracture/ occipital fracture/pneumocephalus/pneumothorax/splenic injury	Unstable/stabled with resuscitation	+	Intraperitoneal blood/grade 3 splenic injury/grade 1 liver laceration/contrast blush in subhepatic space	Open surgery/ligation	None	Survived
5	Fallon et al. (2013)^9^	Male/8	Falling onto the handlebar of a bike	The anterior division of the right hepatic artery	Liver laceration	Unstable/stabled with resuscitation	+	4 cm liver laceration/right anterior branch division hepatic artery transection/active contrast extravasation/free fluid in the abdomen	Radiologic/transarterial micro‐coil embolization	Biloma/pleural effusion	Survived
6	Dullet et al. (2017)^10^	N/A/50	T‐bone motor accident	Left hepatic artery	Left gastric artery cutoff/splenic contusion	Unstable	N/A	Subtle grade 1/grade 2 contusion of superior splenic pole/no definitive sign of active hemorrhage/free fluid in perihepatic and perisplenic area	Radiologic/detachable coil embolization	None	Survived
7	Danial et al. (2020)^7^	Male/27	Falling from a height of 5 m	Common hepatic artery	None	Unstable	+	CT was not performed due to hemodynamic instability	Open surgery/end‐to‐end anastomosis	None	Survived
8	Naseri et al. (2024)	Male/25	Car versus car accident	Proper hepatic artery	SMV injury	Stable/eventually became unstable	_	Vascular injury in porta hepatis/moderate free fluid under the liver and around the spleen	Open surgery/ligation	Iatrogenic injury to the splenic capsule and pancreas tail	Deceased
Overall; *N* = 8		Male: 5 (62.5%) N/A: 3 (37.5%) 25.5 ± 14.9 years		Left hepatic artery: 5 (62.5%) The anterior division of the right hepatic artery: 1 (12.5%) Common hepatic artery: 1 (12.5%) Proper hepatic artery: 1 (12.5%)	Present: 5 (62.5%) Absent: 1 (12.5%) N/A: 2 (25%)	Unstable: 5 (62.5%) Stable: 1 (12.5%) N/A: 2 (25%)	(+): 5 (62.5%) (−): 1 (12.5%) N/A: 2 (25%)		Open surgery: 6 (75%) Radiological: 2 (25%)		Survived: 4 (50%) Deceased: 1 (12.5%) N/A: 3 (37.5%)

Abbreviations: cm, centimeter; CT, computed tomography; FAST, focused assessment sonography in trauma; *N*, number; N/A, not available.

^a^ Graham et al. reported two cases of left hepatic artery avulsion. Unfortunately, we couldn't access the full article.

### 3.2 Diagnostic and management challenges

The high mortality rate in visceral vascular injuries demands proper management and an appropriate diagnostic method to prevent devastating outcomes. Salim et al. highlighted that patients involved in high‐energy trauma who showed no obvious signs of trauma on the initial physical examination could eventually exhibit abnormal findings when trauma pan‐scans were conducted.^13^ These unexpected findings might lead to modifications in the initial treatment plan. E‐FAST is one of the primary evaluating tools, with a sensitivity of 33.3% in stable and 48% in unstable patients, for assessing blunt trauma patients in many centers, considering its availability and rapidity.^14^, ^15^ Although its sensitivity questions its value in managing blunt abdominal trauma patients and explains the negative result in our case regardless of internal bleeding, it has been demonstrated that it is essential to perform E‐FAST since it can provide valuable prognostic data.^16^ Therefore, the index of suspicion in surgeons should be high in cases of such trauma, even in hemodynamically stable patients and the absence of free fluids in E‐FAST.

In comparison with E‐FAST, a CT scan is a more favored method for detecting free abdominal fluid, hematoma, pseudoaneurysms, vessel occlusions, organ contusions, and parenchymal damage as a result of its high sensitivity and specificity.^17^, ^18^, ^19^ Our patient was hemodynamically stable and had a negative E‐FAST result at the time of admission. Due to the injury's high‐energy mechanism, the patient required further observations and CT scanning but did not initially meet the criteria for an immediate CT. Consequently, the abdominopelvic CT was postponed for 2 h, but the free fluid presence was confirmed upon completion of the scan. Extravasation of intravenous contrast in the porta hepatis area was identified in the CT scan; therefore, the patient was transferred directly to the operating room for further exploration of the source of bleeding.

To attain a sufficient visualization for exposing the entire abdominal aorta through left medial visceral rotation, also known as the Mattox maneuver, our patient sustained unfortunate damage to both the splenic capsule and pancreas tail. It has been stated that this procedure may result in damage to the spleen, left kidney, and accessory vessels originating from the kidney.^2^ The risk of iatrogenic spleen injury during medial visceral rotation is calculated to be up to 21.3% and can significantly increase to 60% in emergency procedures.^20^ Still, the importance of this technique in facilitating the management of bleeding compensates for its consequences.^21^ The urgent need for exploratory laparotomy and the unfortunate aforementioned damages led the attending surgeon to perform a splenectomy with distal pancreatectomy to limit the impact of the damage. Therefore, surgeons should handle the spleen gently and exercise caution to avoid any direct pressure.

### 3.3 Surgical management options

Management of hepatic artery injury can be obtained by the available approaches, such as end‐to‐end anastomosis or lateral arteriorrhaphy with or without vein patch, graft interposition, vascular transposition, embolization by endovascular stenting, and ligation.^12^, ^22^, ^23^ The current trend is toward non‐operative management in hemodynamically stable patients after resuscitation to minimize surgical stress and complications.^9^, ^10^ Unfortunately, in the setting of hepatic artery transection, massive blood loss causes inevitable hemodynamic instability in most patients and limits management choices.^12^ Based on the work of Jurkovich et al. on portal triad injuries, arterial ligation benefits the patients when blood replacement is implicated, as primary repair is time‐consuming.^5^ Yet, Danial et al. demonstrated that end‐to‐end anastomosis is advantageous in the presence of isolated hepatic artery injury.^7^ Overall, in the absence of a specific guideline regarding decision‐making, the patient's hemodynamic status, associated injuries, and surgical complexity are the key factors for devising a treatment plan.^2^ Our patient's hemodynamic condition did not stabilize with the initial resuscitation; hence, open surgery was mandatory. Considering the accompanying SMV injury and massive blood loss, our surgeon decided that arterial ligation would be the appropriate decision.

In surgery, to ensure patient survival, the patient's physiology must be aggressively restored, and the only acceptable concept is DCS.^24^, ^25^ Despite the essential determinants of the need for this protocol, deciding when DCS is indicated requires superb clinical and surgical judgment.^26^, ^27^ Hypothermia, acidosis, and/or coagulopathy are the essential, most commonly defined indications in the literature, with an associated massive hemorrhage for facilitating decision‐making.^26^, ^28^ Nevertheless, for optimal patient outcomes, many surgeons believe that the decision to perform DCS should be made before the patient develops physiological exhaustion.^29^ Our patient had an initial temperature of 37.7°C, received five units of PC during his operation, and his intra‐operative blood gas showed a pH of 7.14. These clues are likely associated with an increasing possibility of the potential risks of uncontrollable hemorrhage in the patient who had already been given large volumes of unbalanced ratios of PC and crystalloid fluid. Therefore, the attending surgeon was compelled to perform DCS. However, the patient did not respond properly.

### 3.4 Outcomes

Unfortunately, we could not save this patient; he died due to massive blood loss and subsequent cardiac arrest. We believe that the main obstacle in this case was the initial stability, GCS of 15, and negative FAST, which concealed the presence of vascular injuries and delayed the diagnosis. The rarity of hepatic artery transection due to blunt trauma also disadvantaged the patient since it was inconspicuous for our surgeons. It is possible that an early diagnosis could have resulted in better management of the situation and prevented the patient's unfortunate death. Rendering this situation is particularly challenging to manage due to its rarity, thus necessitating the expertise of competent surgeons. We presented this patient, aiming to contribute to the perfection of the current knowledge on the management of such cases. We believe that by informing surgeons, averting such adverse outcomes is achievable, and careful monitoring of these patients is required for early identification, which could lead to successful management.

In addition to our management, it is worth mentioning that following the ligation of PHA and in the absence of arterial blood flow, the patient may experience further hepatic injuries, especially to hepatic ducts which can also lead to irreversible liver failure or hepatic parenchymal compromise and delayed biliary stricture.^30^, ^31^ This mandates the need for frequent and long‐term biliary intervention, and early restoration of blood fellow to liver by an experienced hepatobiliary surgeon if possible. As a result, surgeons should be aware of this impact before and after making such a decision.

## 4 CONCLUSION

In this report, we described a patient who sustained PHA avulsion from blunt abdominal trauma. Early diagnosis is vital in managing patients with this rare yet life‐endangering injury, as hepatic artery avulsion following blunt abdominal trauma can present with initially normal physical examinations, potentially delaying identification and intervention. To our knowledge, this type of injury has not been reported in previous literature. Due to the uncommon nature of this case, successful management hinges on the expertise of qualified surgeons.

## AUTHOR CONTRIBUTIONS

**Reyhaneh Naseri:** Writing – original draft; writing – review and editing. **Fatemeh Alishavandi:** Writing – original draft; writing – review and editing. **Mohammad Sadegh Rajabian:** Data curation; writing – review and editing. **Hossein Abdolrahimzadeh Fard:** Supervision; writing – review and editing.

## FUNDING INFORMATION

No external funding was received for this project.

## CONFLICT OF INTEREST STATEMENT

The authors declare that they have no competing interests.

## ETHICS STATEMENT

All protocols were approved by the Ethics Committee of the Shiraz University of Medical Science. Also, the study was carried out in accordance with the relevant guidelines and regulations and the Declaration of Helsinki. Written informed consent was obtained from the guardian for the publication of any potentially identifiable images or data included in this article. The patient's information was anonymized prior to the study, and confidentiality was assured by the researchers.

## CONSENT

Written informed consent for publication of the patient's clinical details and pathologic images was obtained from the patient's guardian. A copy of the consent form is available for review by the Editor of this journal. Written informed consent was obtained from the patient to publish this report in accordance with the journal's patient consent policy.

## Data Availability

All data regarding this study has been reported in the manuscript. Please contact the corresponding author if you are interested in any further information.
